# Sex, symptom, and premorbid social functioning associated with perceptual organization dysfunction in schizophrenia

**DOI:** 10.3389/fpsyg.2013.00547

**Published:** 2013-08-27

**Authors:** Jamie Joseph, Grace Bae, Steven M. Silverstein

**Affiliations:** ^1^Rutgers Biomedical and Health Sciences, Rutgers University, Graduate Program in NeurosciencePiscataway, NJ, USA; ^2^Rutgers Biomedical and Health Sciences, Rutgers University Behavioral Health Care, Division of Schizophrenia ResearchPiscataway, NJ, USA; ^3^Rutgers Biomedical and Health Sciences, Robert Wood Johnson Medical School, Department of PsychiatryPiscataway, NJ, USA

**Keywords:** schizophrenia, visual perceptual organization, sex differences, premorbid social sexual functioning, disorganized symptoms

## Abstract

Impairments in visual perceptual organization abilities are a repeatedly observed cognitive deficit in schizophrenia. These impairments have been found to be most prominent among patients with histories of poor premorbid social functioning, disorganized symptoms, and poor clinical outcomes. Despite the demonstration of significant sex differences for these clinical factors in schizophrenia, the extent of sex differences for visual perceptual organization in schizophrenia is unknown. Therefore, we investigated the extent to which previously known correlates (premorbid social sexual functioning and disorganized symptoms) and a novel factor (participant sex) accounted for performance on two perceptual organization tasks (contour integration and Ebbinghaus illusion) that have previously demonstrated sensitivity to schizophrenia. We also determined the relative degree to which each of these factors predicted task scores over and above the others. Schizophrenia patients (*N* = 109, 43 females) from different levels of care were ascertained. Female patients demonstrated higher contour integration scores, but lower performance on the context sensitivity index of the Ebbinghaus illusion, compared to males. Contour integration performance was significantly associated with poorer premorbid adolescent social sexual functioning and higher levels of disorganized symptoms, supporting past results that indicate a relationship among poor premorbid social sexual functioning, disorganized symptoms, and visual perceptual abnormalities in schizophrenia. However, analyses of Ebbinghaus illusion performance suggests there is a complex relationship among patient sex, clinical factors and perceptual abilities with relatively intact bottom–up grouping processes in females, but greater problems, compared to males with more top–down mediated context sensitivity. Therefore, sex differences may be an important consideration for future studies of visual perceptual organization in schizophrenia.

## Introduction

Schizophrenia is a serious psychiatric condition characterized by significant cognitive and perceptual impairments. However, there is a great deal of heterogeneity across patients in terms of types of impairments, levels of severity across impairments (Heinrichs and Zakzanis, 1998; Heinrichs, 2001) and functional outcomes (Green, 1996; Silverstein et al., 1998; Green et al., 2000). One phenomenon that has shown potential for reliably identifying subgroups of patients is reduced visual perceptual organization. This is defined as the processes involved in binding stimulus features into meaningful patterns, groupings or object representations. Perceptual organization impairments have been proposed to be part of a widespread impairment in binding related features, and coordinating cognitive activity, across space and time, in schizophrenia (Phillips and Silverstein, 2003, 2013). Impaired cognitive coordination is also thought to be the core deficit in the disorganized syndrome of schizophrenia (Phillips and Silverstein, 2003), based on past findings of significant relationships between perceptual organizations deficits, and increased cognitive and/or behavioral disorganization but not positive or negative symptoms (Place and Gilmore, 1980; Knight, 1984; Uhlhaas and Silverstein, 2005; Silverstein and Keane, 2011).

Perceptual organization impairments may also be relevant for understanding heterogeneity in the developmental course of schizophrenia as they have been found in patients with histories of poor, but not those with good, premorbid social functioning. Poor premorbid social functioning has been found to increase the risk for the emergence of disorganized symptoms (Wickham et al., 2001; Schenkel et al., 2005) and both premorbid functioning and disorganized symptoms are related to poorer prognosis, which is also a clinical correlate of impaired perceptual organization (Silverstein et al., 1998, 2006; Uhlhaas et al., 2006b).

A limitation of many perceptual studies in schizophrenia is that few account for sex differences in relation to the significant heterogeneity. However, wide-ranging clinical findings among schizophrenia patients suggest that sex differences should be considered in studies of the disorder. A number of reports theorize an influence of sex in the development of schizophrenia pathogenesis (Goldstein et al., 1990; Seeman and Lang, 1990; Hoff and Kremen, 2002; Walker et al., 2002; Goldberg et al., 2011; Walder et al., 2012, 2013). Specifically, sex differences in schizophrenia have been associated with premorbid functioning (Goldberg et al., 2011; Ochoa et al., 2012), age of onset (Walker et al., 2002; Goldberg et al., 2011; Zhang et al., 2012), symptomatology (Walker et al., 2002; Zhang et al., 2012), relapse rate (Ochoa et al., 2012), cognitive ability (Goldstein et al., 1998; Weiser et al., 2000; Hoff and Kremen, 2002), treatment response (Angermeyer et al., 1990; Buchsbaum, 1992; Buchsbaum et al., 1992; Carpiniello et al., 2012) and substance abuse (Mahoney et al., 2010).

Although perceptual organization abnormalities have been linked to factors that are suggested to vary by sex in schizophrenia (e.g., premorbid social functioning, treatment response), there have been no reports of sex differences in perceptual organization impairments in schizophrenia. There is reason to believe that sex differences might exist, since sex differences have been reported in non-clinical samples on tests of perceptual organization (Phillips et al., 2004). Therefore, the goal of this study was to determine whether sex differences on tests of perceptual organization exist in a sample of schizophrenia patients, and the extent to which these are related to other aspects of heterogeneity that have been previously linked to both variables (e.g., premorbid social functioning, disorganized symptoms).

## Materials and methods

### Participants

The study was approved by the University of Medicine and Dentistry (UMDNJ)—Robert Wood Johnson Medical School Institutional Review Board and written consent was obtained for all study participants. The study sample ascertained consisted of 66 male and 43 female patients who met DSM IV-TR (American Psychiatric Association, 2000) criteria for schizophrenia or schizoaffective disorder. Individuals with current substance abuse, mental retardation, neurological disorders, or other primary psychiatric disorders were excluded from the study. To avoid confounds due to moderate attentional deficits, patients with poor catch trial performance, reflecting significant inattention to the tasks (*n* = 22 see Section Data Analyses), were excluded. Included patients (52 males, 35 females) ranged in age from 24 to 64 (*M* = 46.62, *SD* = 10.70).

Patients were recruited from three levels of care within a vertically integrated system (Smith et al., 1999): (1) acute partial hospital (most recent inpatient discharge was within the past 6 months and includes full daily structure and treatment); (2) extended partial hospital (where the last inpatient discharge occurred over 6 months ago, but daily treatment and structure are still required); and (3) outpatient (where last inpatient discharge was over 2 years ago and patients are considered to be clinically stable requiring bi-weekly or monthly visits to psychiatric care providers).

### Clinical assessments

Psychiatric diagnoses for all participants were determined with the Diagnostic Interview for Genetic Studies (DIGS) (Nurnberger et al., 1994) and confirmed via review of UMDNJ-UBHC medical records in order to determine study eligibility. Symptoms occurring 2 weeks prior to testing were assessed using the Positive and Negative Syndrome Scale (PANSS) (Kay et al., 1987). Syndromes were then analyzed based on a five factor model (Lindenmayer et al., 1994) which includes positive, negative, cognitive, excitement, and depression factors.

As disorganization has been shown to be a strong correlate of both perceptual disorganization and premorbid social functioning, disorganized symptoms were characterized in two ways. The first method is similar to a strategy used in one of our previous studies where significant differences in perceptual organization ability were observed when patients were dichotomized, based on the PANSS conceptual disorganization item score (Uhlhaas et al., 2006a), into either a disorganized or non-disorganized group. For the present study, participants with a score of 2 or lower (i.e., within the normal range) were considered the non-disorganized schizophrenia group (*n* = 32) and participants with a score of 3 (mild symptoms) or greater comprised the disorganized schizophrenia group (*n* = 53). These groups were then compared using *t*-tests. For all other analyses, disorganized symptoms were analyzed based on a separate disorganization factor identified by Cuesta and Peralta (1995) that is not part of the original PANSS. This factor includes the PANSS items of conceptual disorganization, and poor attention, as well as the inappropriate affect item developed by Cuesta and Peralta.

Psychosocial adjustment and academic development were evaluated using the Premorbid Adjustment Scale (PAS) (Cannon-Spoor et al., 1982). Each item was scored on a scale of 0 (good) to 6 (poor) from early childhood until 1 year prior to the onset of first psychotic symptoms, or 1 year prior to first psychiatric hospitalization if exact age of onset could not be determined. Scores on 5 domains of functioning were calculated: social withdrawal, peer relationships, scholastic performance, school adaptation, social-sexual aspects of life and an overall mean score (Cannon-Spoor et al., 1982). Much prior literature has assessed the association of perceptual deficits and premorbid functioning by using the Phillips or Zigler-Phillips scale of premorbid adjustment (Zigler, 1961). These scales primarily comprise questions about friendships, dating, and marriage. They correspond closely to the premorbid social sexual functioning factor of the PAS, a more current and widely used scale. Because: (1) our primary interest was in premorbid social-sexual functioning, which we have assessed in past studies and which has been found to correlate inversely with perceptual organization ability; and (2) there are no data to suggest that other aspects of premorbid functioning (e.g., academic functioning) would or should be related to perceptual organization, only scores on the social sexual subscale of the PAS were prioritized for analyses with perceptual indices.

Study participants were taking atypical and/or typical antipsychotic medications with stable medication dosages. Antipsychotic medication dosages were converted to chlorpromazine equivalents based on published standards (Andreasen et al., 2010). Recent and typical tobacco use for was assessed with the Fagerström Test for Nicotine Dependence (Heatherton et al., 1991) to determine the current effects of nicotine use or dependence on perceptual task performance.

The vocabulary subtest of the Shipley Institute of Living Scale (Zachary, 1991) was administered to participants in order to estimate IQ by converting raw scores into a WAIS-R IQ score. A Snellen chart was used to ascertain visual acuity estimates for each eye and then both eyes. Handedness was ascertained with a questionnaire about hand preference for different daily tasks including: writing, throwing, using scissors, etc. Participants were noted as right handed or left handed based on these responses.

### Perceptual organization tasks

#### Contour integration-JOVI task

**Figure 1:**
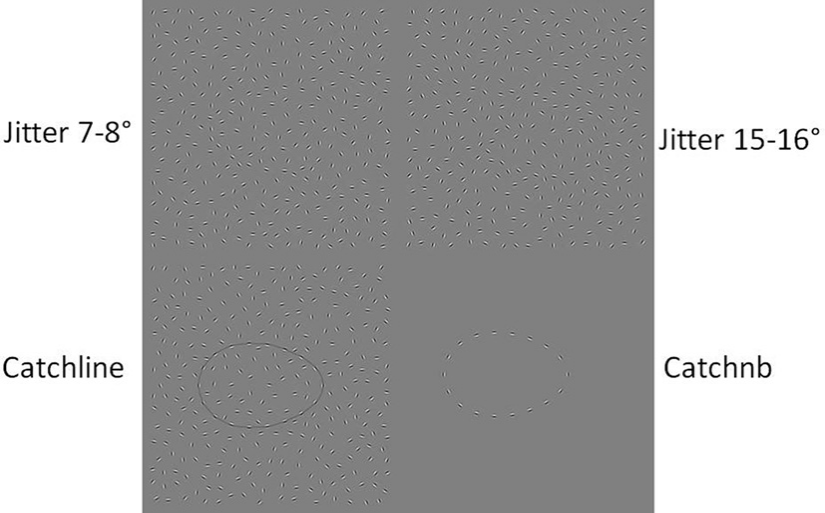
**JOVI task stimuli.** This figure illustrates the stimuli used for the JOVI task. The top **top left** panel of the figure is an example of a lower jitter degree condition presented to participants (7–8°). The **top right** panel of the figure shows the highest jitter degree presented (15–16°). The **bottom left** and **right** panels show the catch stimuli included in each block to account for participant attention level (see text for description of these stimuli).

The Jittered Orientation Visual Integration (JOVI) task is a test of contour integration that determines a participant's ability to integrate Gabor elements into a perceptual whole. Gabor elements are sinusoidal luminance distributions that are Gaussian modulated (Silverstein et al., 2000; Uhlhaas et al., 2005). That is, Gabor elements show lower contrast at the edges compared to the center, and luminance varies from white to black in a gradually alternating fashion (Figure 1). Gabor elements are considered to activate orientation-selective feature detectors in the primary visual cortex (Angelucci and Bullier, 2003), and are therefore a useful means to study their integration in early visual processing.

The stimuli presented for this task are based on our recent study of contour integration in schizophrenia (Silverstein et al., 2012b). Participants were shown static Gabor elements arranged in an oblong shape forming a contour embedded in a display of randomly oriented Gabor elements. The degree of orientation jitter of contour Gabor elements varied across six conditions (±0°, 7–8°, 9–10°, 11–12°, 13–14°, 15–16°), and this manipulation placed increasing degrees of burden on perceptual organization processes (i.e., at higher jitter levels, the correlations between adjacent element orientations become weaker, contour smoothness is increasingly disrupted, and contour perception becomes more difficult). For all stimuli, the ratio of the density of adjacent background elements to the density of adjacent contour elements was 0.9. Because adjacent background elements were, on average, closer together than adjacent contour elements, perceptual organization processes used to process contours are independent of density cues for this task (Silverstein et al., 2000).

All JOVI stimuli trials were presented for 2 s followed by a 1 s inter stimulus interval during which responses were no longer recorded. There were 48 stimulus trials per jitter condition which were presented in blocks of 12 trials by condition. In addition, two types of catch stimuli (no errors expected) using 0° jitter were administered during each block to assess for attention lapses. One catch trial type had curved lines drawn through the contours (to eliminate the need for perceptual organization), and the other contained contour elements without any background elements (to remove effects of noise). The JOVI is a symmetric 1-alternative forced choice task where subjects responded whether the narrow end of the oblong contour was pointing left or right (Figure 1). The task and stimuli patterns were created using E-prime (Psychology Software Tools, Pittsburgh, PA).

#### Ebbinghaus illusion task

**Figure 2:**
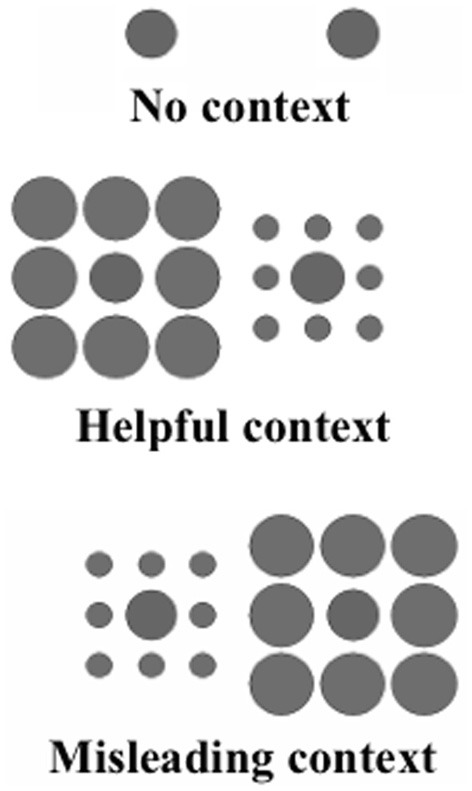
**Ebbinghaus illusion stimuli.** This figure illustrates the stimuli used for the Ebbinghaus illusion task. The **top** panel shows an example of the no outer context trials, the **middle** panel shows an example of the helpful context trials and the **bottom** panel is an example of the misleading context trials. In all three panels, the left center circle is 2 pixels smaller than the right center circle.

The Ebbinghaus illusion assesses integration of non-target information during target perception, and the resulting change in size perception of the target is thought to result from size constancy (Doherty et al., 2008, 2010). In this task (Figure 2), the perceived size of center circles is modified by the presence of outer context circles: typically, larger context circles make the center circle appear smaller than its actual size, while smaller context circles lead to an enlarged appearance of the center circle (Uhlhaas et al., 2006a).

The stimuli used for this task were developed by Phillips et al. (2004), Doherty et al. (2010). Participants were shown two black circles presented on a white background with one center circle 100 pixels in diameter and the second center circle varying by 2, 6, 10, 14, or 18 pixels (This corresponded to one center circle having 2.67° of visual angle in diameter, with the other center circle 0.05, 0.16, 0.27, 0.37, or 0.48° larger or smaller). These center circles were shown with three different surrounding contexts: no outer context, misleading context (outer context circles should impair accurate inner circle size discrimination) and helpful context (outer context should aid accurate inner circle size discrimination). The no context condition had 96 trials: 32 at the smallest size difference and 16 at all other size differences. The misleading context condition had 80 trials: 16 at each level of inner circle size difference. The Helpful context condition had 16 trials, all at a 2 pixel difference. Stimuli were presented for 2 s with 200 ms inter stimulus interval, in a random order. Subjects responded whether the left center circle or right center circle was larger (see Figure 2). The task and stimuli were created using C++.

### Data analyses

All data were analyzed using IBM SPSS Statistics 19. The demographic, clinical and perceptual data were compared between male and female participants using independent samples *t*-tests or χ^2^ square tests to analyze categorical variables (Table 1). Spearman correlations were performed to examine the associations among demographic factors, PAS factors, PANSS factors and perceptual scores (Tables 2–4).

**Table 1:** **Demographic clinical and perceptual characteristics based on participant sex**.

**Demographic clinical and perceptual factors**	**Female (*n* = 35)**	**Male (*n* = 52)**	***p***	***d***
Age	47.54 (10.86)	46.00 (10.66)	0.513	0.14
Estimated age of onset	22.22 (7.76)	22.14 (6.23)	0.960	0.01
Participant education level	12.44 (2.15)	12.50 (2.58)	0.914	−0.03
Mother education level	11.90 (2.90)	12.51 (2.42)	0.330	−0.23
Father education level	12.75 (3.83)	13.36 (2.66)	0.461	−0.18
Shipley vocabulary subtest	88.89 (12.52)	90.50 (14.14)	0.586	−0.12
Total chlorpromazine equivalent	522.84 (367.83)	490.05 (340.01)	0.683	0.09
Total JOVI (contour integration) task score	181.23 (36.02)	159.17 (41.43)	0.012	0.57
Ebbinghaus task index score	1.20 (13.03)	8.13 (10.35)	0.010	−0.58
PAS overall mean	2.49 (0.66)	2.73 (0.83)	0.177	−0.32
PAS adolescent only social sexual factor	1.16 (1.35)	2.65 (1.94)	<0.001^***^	−0.89
PAS social sexual factor	3.81 (1.98)	5.37 (4.29)	0.031^*^	−0.47
PAS social withdrawal factor	9.88 (4.51)	10.92 (4.62)	0.319	−0.23
PAS peer relation factor	7.97 (3.81)	8.47 (3.28)	0.531	−0.14
PAS scholastic factor	7.81 (3.70)	8.80 (3.63)	0.240	−0.27
PAS school adaptation factor	6.13 (3.27)	7.94 (3.16)	0.015^*^	−0.56
PANSS cuesta and peralta disorganization factor Score	7.09 (1.96)	7.80 (2.38)	0.148	−0.33
PANSS cognitive factor	13.09 (2.73)	14.14 (3.48)	0.138	−0.34
PANSS negative factor	15.69 (3.99)	16.56 (4.31)	0.345	−0.21
PANSS positive factor	11.06 (2.81)	11.38 (2.84)	0.605	−0.11
PANSS depression factor	14.46 (4.67)	14.12 (3.21)	0.694	0.08
PANSS excitement factor	10.14 (2.34)	9.70 (1.81)	0.328	0.21
Race African American/Caucasian^χ^	14/21	26/26	0.388	0.09^φ^
Handedness left/right^χ^	7/28	5/47	0.211	0.15^φ^
Primary psychiatric diagnosis schizophrenia/schizoaffective^χ^	15/18	37/13	0.011^*^	0.29^φ^
Current smoker YES/NO^χ^	20/15	24/28	0.384	0.10^φ^
Current antipsychotic medications typical/atypical/both^χ^	5/27/3	5/40/7	0.660	0.10^φ^

χ chi square test;

* p < 0.05;

*** p < 0.001;

φ phi correlation coefficient.

**Table 2:** **Spearman correlations of demographic factors and perceptual task indices**.

**Demographic factor**	**JOVI index**		**Ebbinghaus index**	
	***r***	***p***	***r***	***p***
Age	−0.095	0.379	0.106	0.325
Estimated age of onset	0.024	0.827	0.278	0.001^**^
Participant education level	−0.106	0.328	0.215	0.044^*^
Mother education level	−0.138	0.246	0.187	0.113
Father education level	−0.117	0.363	0.105	0.415
Shipley vocabulary subtest score	0.125	0.247	0.246	0.021^*^
Total chlorpromazine equivalent	−0.039	0.725	0.006	0.957
Race	−0.071	0.511	0.068	0.531
Sex	−0.259	0.015^*^	0.274	0.010^*^
Handedness	−0.203	0.057	0.086	0.427
Primary psychiatric diagnosis	−0.137	0.213	0.038	0.732
Current smoker	−0.117	0.278	−0.214	0.047^*^
Current antipsychotic medications	0.084	0.437	−0.023	0.834

* p < 0.05;

** p < 0.01.

**Table 3:** **Spearman correlations of PANSS factors and perceptual organization task indices**.

**PANSS factor (*n* = 85)**	**JOVI index**		**Ebbinghaus index**	
	***r***	***p***	***r***	***p***
Cuesta & Peralta disorganization	−0.333	0.002^**^	−0.001	0.991
Cognitive	−0.379	<0.001^***^	−0.172	0.116
Negative	−0.071	0.518	0.236	0.030^*^
Positive	−0.075	0.495	0.157	0.152
Depression	0.076	0.492	0.030	0.785
Excitement	0.002	0.985	−0.020	0.857

* p < 0.05;

** p < 0.01;

*** p < 0.001.

**Table 4:** **Spearman correlations of premorbid adjustment scale factors and perceptual organization task indices**.

**Premorbid adjustment scale factor (***n*** = **81**)**	**JOVI index**		**Ebbinghaus index**	
	***r***	***p***	***r***	***p***
Overall mean	−0.003	0.980	−0.228	0.040^*^
Adolescent only social sexual factor	−0.240	0.031^*^	0.258	0.020^*^
Social sexual factor	−0.181	0.105	0.195	0.081
Social withdrawal factor	0.017	0.882	0.019	0.867
Peer relation factor	−0.077	0.495	0.061	0.590
Scholastic factor	0.123	0.275	−0.223	0.045^*^
School adaptation factor	−0.049	0.665	−0.225	0.043^*^

* p < 0.05.

For multiple regression analyses, in cases where correlational analyses identified significant predictors of task performance that were predicted *a priori* (e.g., poor premorbid social functioning, disorganized symptoms), hierarchical regression analyses were employed in order to determine the extent to which each significant predictor accounted for variance in the dependent variable over and above that of other predictors, in addition to which variable alone accounted for the greatest proportion of variance in test score. In cases where correlational analyses indicated significant predictors that were not predicted *a priori*, stepwise regression was used to determine the descending order in which these predictors accounted for variance in the dependent variable.

For the contour integration (JOVI) task, the mean score across all jitter conditions was used as the performance index since a previous study suggested higher test-retest reliability for the overall mean score compared to threshold values (Silverstein et al., 2012a). Study participants with JOVI catch trial scores below 90% were excluded from all data analyses (*n* = 22). For the Ebbinghaus illusion task, a difference score index was computed: [(Helpful Context 2 Pixel Condition-No Outer Context 2 Pixel Condition)—(Misleading Context 2 Pixel Condition-No Outer Context 2 Pixel Condition)], which reflected overall context sensitivity.

## Results

### Demographic clinical and perceptual differences based on participant sex

Female and male participant demographic, PAS, PANSS and perceptual task score differences are shown in Table 1.

### Demographic factors and perceptual task performance

The correlations between demographic factors and perceptual task indices are shown in Table 2.

### PANSS symptom factors and perceptual task performance

Spearman correlations between PANSS symptom factor scores and perceptual task performance are listed in Table 3. In terms of the dichotomy between disorganized and non-disorganized patients; only Total JOVI scores [non-disorganized group: *M* = 182.88, *SD* = 36.00, disorganized group: *M* = 162.07, *SD* = 38.67; *t*_(83)_ = 2.47, *p* = 0.016, *d* = 0.56] and not Ebbinghaus index scores [non-disorganized group: *M* = 6.06, *SD* = 11.88; disorganized group: *M* = 5.17, *SD* = 11.95; *t*_(84)_ = 0.34, *p* = 0.737, *d* = 0.07] were significantly different.

### PAS factors and perceptual task performance

Spearman correlations between PANSS symptom factor scores and perceptual task performance are listed in Table 4.

### Inter task correlations are not significant

The correlation between the JOVI and Ebbinghaus performance indices was not statistically significant: *r*_(87)_ = 0.024, *p* = 0.822.

### Hierarchical regression examining significant correlates of contour integration performance

The set of factors that were significantly correlated with lower JOVI scores included: male sex, poor premorbid adolescent social sexual functioning, higher PANSS cognitive factor scores and higher Cuesta and Peralta disorganized factor scores. Two of these factors, poor premorbid social functioning and disorganized symptoms, were previously associated with contour integration performance (Uhlhaas et al., 2006a). The PANSS cognitive factor was excluded to avoid multicollinearity (i.e., *r* > 0.795) due to a strong correlation with disorganized factor symptoms *r*_(86)_ = 0.887, *p* < 0.001.

Hierarchical regression analyses were conducted to answer 3 distinct questions: (1) which variable, alone, accounted for the most variance in JOVI score; (2) which variable, after all other variables were entered at prior steps, accounted for the most additional variance in JOVI score; and (3) given the study's focus on sex differences, to what extent did sex differences account for additional variance in JOVI score after all other variables were entered at a prior step. The factor that, alone, explained the most variance in JOVI performance was disorganized symptoms: *B* = −5.37, *SE B* = 1.89, β = −0.30, *R*^2^ = 0.092, *F* = 8.01, *p* = 0.006. While participant sex (*B* = −12.19, *SE B* = 9.27, β = −0.15, *t* = −1.32, *p* = 0.192) premorbid adolescent social sexual functioning (*B* = −2.98, *SE B* = 2.43, β = −0.14, *t* = −1.23, *p* = 0.224) and disorganized symptom factor scores (*B* = −4.32, *SE B* = 1.91, β = −0.24, *t* = −2.26, *p* = 0.026) contributed, as a set, to total JOVI score (*R*^2^ = 0.148, *F* = 4.46, *p* = 0.006), disorganized factor scores still accounted for unique variance over and above these other variables: *R*^2^ change = 0.055, *F*_(1, 77)_ = 4.96, *p* = 0.029, and this was the largest increase of one variable over and above any of the others. Examination of the effect of sex (entered at step 2) over and above other variables (entered as a set at step 1) indicated a non-significant additive effect: *R*^2^ change = 0.019, *F* = 1.73, *p* = 0.192 (*B*, *SE B*, and β-values are the same as above).

### Stepwise regression examining significant correlates of ebbinghaus illusion performance

The set of factors that were correlated with Ebbinghaus illusion performance were patient sex, PANSS negative symptom factor score, Shipley subtest score, premorbid social sexual adolescent functioning, premorbid scholastic factor performance, premorbid school adaptation factor performance, age of onset and current smoking status. Since, except for the PAS social sexual functioning variable, there were no *a priori* hypotheses as to how these factors would contribute to variance in Ebbinghaus illusion performance, a stepwise regression was performed. The factors were entered, by the stepwise regression algorithm, in the following order: sex, age of onset, premorbid social sexual adolescent functioning, PANSS negative symptom factor, PAS school adaptation factor, PAS scholastic factor and current smoking status. The stepwise analysis indicated that the combination of these factors accounted for significant variance in the Ebbinghaus performance index: *R*^2^ = 0.33, *F* = 9.04, *p* < 0.001. Participant sex alone made a significant contribution to variance in Ebbinghaus performance: *B* = −9.11, *SE B* = 2.56, β = −0.37, *R*^2^ = 0.14, *F* = 12.26, *p* = 0.001. In addition, even after all of the other statistically significant predictor variables were entered as a set in block 1 of a second regression analysis, sex continued to be a significant predictor when entered alone in block 2: *B* = 9.34, *SE B* = 2.59, β = 0.38, *R*^2^ change = 0.11, *F* = 12.97, *p* < 0.001.

## Discussion

This study replicated previously reported findings that impaired contour integration was associated with poorer premorbid social functioning and increased disorganization symptoms (Silverstein et al., 2000; Schenkel et al., 2005; Uhlhaas et al., 2005). In the current study, female gender was associated with higher contour integration scores compared to males in a chronic schizophrenia population that included many patients who were sufficiently disabled so as to require daily partial hospital treatment. However, our hierarchical regression results indicated that level of clinical disorganization was a better predictor of impaired contour integration performance than participant sex or premorbid social sexual adolescent functioning. Of note, using a 70% JOVI catch trial cutoff as an exclusion criterion, as opposed to 90%, premorbid social sexual functioning was the stronger predictor. This suggests that the sex differences observed on contour integration performance are mostly in agreement with the hypothesis of a greater neurodevelopmental basis in males with schizophrenia (Goldstein et al., 1998), and with gender differences in premorbid functioning (Goldberg et al., 2011; Ochoa et al., 2012), and neurocognitive task performance (Hoff and Kremen, 2002; Johnson et al., 2005) which tends to be poorer in males. The slight difference in results between analyses using a 70 vs. 90% threshold for JOVI catch trial performance (i.e., 30 vs. 10% attention lapse errors) may also account for the very recent finding of a lack of a sex difference among schizophrenia patients on the JOVI (Strauss et al., 2013). The sample in the Strauss et al. study consisted mostly of clinically stable outpatients who would be expected to have relatively few problems with attention lapses (i.e., they would be more similar to our patients who had fewer than 10% lapse errors than to those with up to 30% lapse errors). Therefore, it may be that sex differences are stronger when more severely ill patients are included in the sample and visual processing impairment is expressed to a greater degree, suggesting an interaction between sex, illness phase, and perception that is in need of further examination.

Factors such as premorbid social functioning and disorganized symptoms have been shown to predict a poor outcome in schizophrenia spectrum populations (Zigler, 1961; Strauss and Carpenter, 1972; Knight et al., 1979; Bearden et al., 2011). Whether these relationships reflect a specific patient subtype or simply strong relationships between selected variables remains an open question. However, our data support past hypotheses that a group of patients characterized by these factors and abnormal perceptual organization exists, and this suggests the importance of including perceptual, developmental, symptom, and gender variables in future studies to characterize heterogeneity in perceptual functioning, and perhaps other aspects of cognition, in schizophrenia.

The gender differences on Ebbinghaus illusion task performance have not been previously reported. Moreover, unlike in our past studies with this illusion, task performance was not related to disorganization symptoms. One possible explanation for the different findings is that acutely ill patients were not included in the study, and some perceptual functions are state sensitive in schizophrenia (e.g., Uhlhaas et al., 2005), including performance on the Ebbinghaus illusion (Silverstein et al., 2013). Therefore, it is possible that our outpatient and partial hospital sample did not have sufficient range in Ebbinghaus scores to detect relationships observed in past studies, which included relatively large numbers of inpatients, or wholly inpatient samples. Future studies of the Ebbinghaus illusion in schizophrenia can clarify the extent to which gender is related to task performance as a function of phase of illness by including acutely ill inpatient samples.

The non-significant correlation between the contour integration task and the Ebbinghaus illusion may indicate that these tasks assess different stages of perceptual organization in schizophrenia. A possible interpretation of these findings is that contour integration reflects relatively more bottom–up than top–down contributions to perception [i.e., more stimulus-driven and lower level aspects of perception, including basic binding processes in V1–V4 (Silverstein et al., 2009)], whereas the Ebbinghaus illusion, by incorporating both grouping and size constancy principles, involves a relatively greater influence of top–down factors including memory and past experience interpreting size based on 2D depth cues (Doherty et al., 2010) [see Purves and Lotto, 2011 for an account of the illusion consistent with this hypothesis, and Dima et al. (2009) for data indicating reduced top-down involvement in another task involving experience-based modulation of perception in schizophrenia]. It is possible that the different mechanisms involved in performance on each task can account to some degree for the differences in gender differences in patient performance across the two tasks. However, at present, there is no obvious explanation for why females with schizophrenia would be superior to males in lower-level vision, while males would be more normal on a higher-level visual task. It is also important to note that the direction of gender differences in our study on the Ebbinghaus illusion, with females with schizophrenia being less context sensitive than males, is in contrast to a previously reported finding in a non-psychiatric population (Phillips et al., 2004). Therefore, this suggests a possible interaction of gender, Ebbinghaus illusion task performance and schizophrenia spectrum diagnosis that requires further exploration.

This study has two significant limitations. The first is that multiple comparisons were not controlled for, and therefore the results should be considered as preliminary and require replication. Another limitation is that psychiatric and non-psychiatric control subjects were not included in the study. The tasks used in this study have been used in other studies that included patient and non-psychiatric controls and these groups have shown significantly superior performance compared to schizophrenia, suggesting specificity for the latter (Silverstein and Keane, 2011). However, the inclusion of non-psychiatric controls and other psychiatric populations in future studies will be necessary to determine whether the correlates of perceptual organization performance are specific to schizophrenia.

The results of our study appear to be relevant for future studies assessing gender, premorbid function and perceptual organization in prodromal, at risk, and first episode populations. Since the perceptual organization tasks employed are considered to have well-defined neurodevelopmental trajectories (Kovacs et al., 1999; Kaldy and Kovacs, 2003; Doherty et al., 2010), it would be useful in future studies to compare and consider perceptual organization in early vs. later stages of schizophrenia to determine if impairments are primarily accounted for by altered maturation of perceptual organization circuitry or illness-related perceptual organization deterioration.

Overall, the present study's findings for contour integration performance support those of earlier smaller studies in indicating that impairment at an early stage of perceptual organization is associated with poorer premorbid social functioning and a tendency toward development of disorganized symptoms. Because other studies indicate that patients with these characteristics also have a poorer response to treatment and a more chronic illness course (Silverstein et al., 1998; Uhlhaas et al., 2006b), the development of greater insight into this group, and the development of new treatment strategies for it, is especially important.

### Conflict of interest statement

The authors declare that the research was conducted in the absence of any commercial or financial relationships that could be construed as a potential conflict of interest.
